# An Analysis of Seventy-Three Appeals

**Published:** 1921-09-17

**Authors:** 


### Approved Societies and Hospitals.

### AN ANALYSIS OF SEVENTY-THREE APPEALS.

In a useful speech, Mr. R. B. Loder, who presided at the quarterly Court of Governors of Northampton General Hospital, reported what had occurred between the hospital and the approved societies. The hospital had sent seventy-three appeals to the approved societies for recompense from their surplus. The nine replies received could be summarised thus:—

The National Deposit Friendly Society had agreed to pay one guinea per week per member treated as an in-patient; the Northampton M.U.O.F. Loyal True Briton Lodge, State Section, had increased from three to ten guineas its annual subscription; the Independent Order of Rechabites, Northampton District, said it was unable to make a grant; the Hearts of Oak Benefit Society Head Office said they would divide the whole surplus for additional cash benefits to members; the Royal Liver Friendly Society would consider the matter; the Thrapston Royal Oak Lodge, Nottingham Order of Oddfellows would increase annual subscription from three to four guineas; the A.O.F. Court Centre of England, Northampton, would give cash benefits to members; the Bedfordshire United Approved Society would subscribe fourteen guineas per annum for five years; and M.U.O.F. Loyal Chandos Lodge, Newport Pagnel, would give ten guineas donation out of ordinary funds. Another speaker said the Prince of Wales Lodge, M.U.O.F., had promised ten guineas for five years out of its surplus. Of the nine societies named by Mr. Loder, three, therefore, refused; one was undecided; and the remaining five promised to help in one way or another. Are the other sixty-four societies to maintain a stony silence?

